# The Reliability and Test-Retest Stability of the Treatment Perception Questionnaire (TPQ) in the Oncology Field: A Pilot Study

**DOI:** 10.2174/1745017902117010324

**Published:** 2021-12-31

**Authors:** Federica Sancassiani, Sara Gambino, Jutta Lindert, Lapo Sali, Irene Pinna, Giulia Origa, Giulia Cossu, Mario Scartozzi, Antonio Preti

**Affiliations:** 1 Department of Medical Sciences and Public Health, University of Cagliari, Cagliari, Italy; 2 University of Applied Sciences Emden/Leer, Emden, Germany; 3 Department of Biomedical, Experimental and Clinical Sciences, University of Florence, Florence, Italy; 4 Department of Neurosciences, University of Turin, Turin, Italy

**Keywords:** Satisfaction with care, Oncology, Validation study, Psychosocial health, Psycho-oncology, Assessment

## Abstract

**Background:**

Patients' satisfaction is an indicator of technical, instrumental, environmental, and interpersonal aspects of care. It shows how much the health service “as a whole organization” meets the patients’ psychosocial expectations and if the health professionals combine their technical competence with relational skills. The Treatment Perception Questionnaire (TPQ) is a brief instrument developed in the United Kingdom for research with substance abuse disorder populations. The present study aimed at evaluating the reliability and test-retest stability of the TPQ Italian translation in a sample of patients with solid and blood cancers.

**Methods:**

The TPQ was administered to 263 people with solid and blood cancers. Test-retest reliability was evaluated in a subgroup of 116 participants who completed the TPQ again after 3 months.

**Results:**

The reliability of TPQ was good. Cronbach’s alpha: 0.83 (95%CI: 0.79-0.86), 0.66 (0.59-0.72), 0.71 (0.65-0.769), respectively, in the total test, and in subscales on “staff perception”, and “program perception”. Test-retest reliability was 0.82 (0.77-0.87). The mean difference between the first and the second assessment was 1.0 (SD = 7.1; 95% CI -0.35 to 2.33). By plotting the differences and the means of the two assessments, 5/116 cases (4.3%) were outside the upper and lower limits of agreement.

**Conclusions:**

This study points out good reliability and test-retest stability of the TPQ in the oncology field. The TPQ can be used to assess variation over time about satisfaction with care in patients with oncological diseases, favoring the identification of unmet patients’ needs about the quality of the service.

## 1 BACKGROUND

The evaluation of satisfaction with care in the oncological field is an important part of the assessment of health care services in terms of service quality and health care system responsiveness [1, 2]. The high frequency of chronic conditions, due also to frequent comorbidity between several diseases and functional impairment resulting from socio-demographic changes that occurred in recent decades, as well as the aging of the population, discloses challenges in public health with a relevant socio-economic dimension. Hence, patients’ satisfaction with care could reveal much information about the impact of diagnostic and treatment effects on their wellbeing [3]. Furthermore, these challenges significantly influence the changes in the relationship between health professionals and patients, showing the role of several psychosocial dimensions in long-lasting mutual interactions in the care setting [4, 5]. Particularly, in the oncology setting, patients' satisfaction with care is an indicator of technical, instrumental, environmental, and interpersonal aspects of care [6-9]. The identification of cancer-specific distress, especially in older patients often suffering from other chronic conditions [10], concerns the health service “as a whole” (*i.e*.: an oncology unit). It is a relevant factor in showing how much the health professionals grab the psychosocial expectations of the patients and if they can combine professional competence with relational and communication skills. The capacity for showing respect, empathy, and interest in patient with cancer, personal and daily life problems are important to establish a good therapeutic alliance and to enhance a good level of motivation and compliance to treatments [6, 11, 12]. Satisfied patients tend to be more confident when using health care services, more compliant with treatments, more likely to maintain a good relationship with health care providers and to endorse the health care service to others’ [13, 14]. For these reasons, patient satisfaction with care is a crucial outcome measure for cancer care [9].

Beyond the oncology field [11], the concept of patient satisfaction with care could be explained as a multidimensional social construct involving several aspects. From a general psychosocial perspective, satisfaction is referred to how and how much an individual evaluates distinct aspects of care received, mainly about an overall assessment of health care as a system. In this way, satisfaction could be considered as a function of the preferences and needs of the patients and the clinical performance provided by healthcare professionals. Finally, satisfaction with care could reflect the balanced evaluation of differences between effective experience in the health service and the patients’ expectations about their needs and desires. So far, considering the patients’ experience with health care services, satisfaction with care has been evaluated in several health contexts, both by quantitative and qualitative methodologies, addressing the use of person-centered measures and the users’ subjectivity for quality improvement in the health care services [1, 15].

There are several patient satisfaction-with-care instruments, including measures related to cancer treatment, as the widely used EORTC-INPATSAT32 [16], designed to assess satisfaction with the in-patient cancer care, or the FAMCARE [17] to evaluate satisfaction among those with advanced cancer, or the Patient Satisfaction with Cancer Care (PSCC) [9], to evaluate the spectrum of cancer-related care from screening to the treatment of diagnosed cancer. However, these scales are long (32 items in the EORTC-INPATSAT32), focused on patient satisfaction with out-patient palliative oncology care (FAMCARE), or measured just one component of patient’s satisfaction with care. Our preference was for an easy tool able to measure the multidimensionality of satisfaction of patients with oncological diseases in the busy setting of the oncology units. For this reason, we chose the Treatment Perception Questionnaire (TPQ) [18, 19]. The present study was set out to evaluate the reliability and the test-retest stability of the Italian translation by Umberto Nizzoli & Claudia Corbelli [20] of the TPQ. The TPQ is a feasible and brief self-report questionnaire comprising of 10 items aimed at evaluating the satisfaction with care of people with abuse of alcohol-related diseases. In patients with addiction, the reliability of the TPQ has been reported to be satisfactory, both as internal coherence and at test-retest stability. The TPQ is expected to measure two main components of patients’ satisfaction with the treatment, as a reflection of the perception of clients towards the nature and extent of their contact with the program staff (“staff perception”) and the perception of clients towards the treatment service and its operation and rules and regulations (“care program perception”). These two dimensions were observed after exploratory factor analysis of the TPQ in a sample of patients with substance use disorders [18, 19]. To our knowledge, the present study is the first in which the TPQ is used to evaluate satisfaction with care in a sample of patients with solid and blood cancers accessing Oncology specialized Units. Particularly, we wanted to assess whether the reliability and test re-test stability of TPQ was good in people with oncological diseases as it was found in patients with addiction.

## 2 METHODS

### 2.1 Sample, Recruitment Procedures and Ethical aspects

The study has been conducted at the Oncology Unit, University-Hospital of Cagliari “Policlinico - Duilio Casula” and at the Hematology Unit and Stem Cell Transplantation Center, Hospital “Businco”, Azienda Ospedaliera “Brotzu”, Cagliari, Italy. Since 2018, all consecutive patients who accessed the two services have been invited to participate in the study, inclusion criterion was: age ≥18 years, male/female, histologic confirmation of malignant neoplasm, receiving active treatment, the signature of written informed consent after a full description of the aims, the procedures, the data protection, and the possibility to terminate the study at any time. The study was approved on September 26, 2018, by the Ethical Committee of the Sardinia Region, Italy, and registered with the number PG/2018/13269. All procedures were carried out under the 1964 Helsinki Declaration and its later amendments.

### 2.2 Instruments

We used the following instruments to assess treatment perception and satisfaction with care, as well as to collect socio-demographics and clinical-oncological data.

Treatment perception and satisfaction with care.

The Treatment Perception Questionnaire (TPQ) was originally developed among patients with substance abuse problems from four treatment services in London [18]. The TPQ is a self-reported questionnaire that included 10 items and takes into account two areas: the first one regards the perception of patients towards the nature and extent of their contact with the health staff (5 items); the second area regards aspects of the care program and its procedures and regulations (5 items). Each item is recorded using a five-point Likert scale (strongly agree – strongly disagree). A principle components analysis of the questionnaire extracted two dimensions (“staff perception” and “care program perception”) which accounted for 51.6% of the variance. The items comprising the two dimensions were internally reliable (α= 0.76 and α= 0.71, respectively), as was the total scale (α= 0.83). Higher scores on the TPQ correspond to higher satisfaction with the treatment’s staff and the program.

### 2.3 Socio-Demographics and Clinical-Oncological Data

To collect socio-demographics and clinical-oncological data we developed a questionnaire including variables such as age, gender, marital and employment status, education level, time of 3-6-9 months follow-up (T0, T1, T2, T3), kind of the oncology service (Day Hospital/Hospital Ward), timing of taking care of the patient at the baseline (first visit, <6 months, 6-12 months, >12 months); solid cancer site (gynecological: uterus, ovaries, cervix; breast; lung; urogenital: prostate, kidney, bladder; gastroenteric: esophagus, stomach, pancreas, colon-rectum; rare cancers: melanoma, sarcoma, cerebral cancer; head; neck), the diffusion of hematological cancer was scored from 1 to 4 considering 1 as a unique localization in one nodal station or extra-nodal; 2 as two or more localizations from the same side of diaphragm, 3 as localizations from both side of diaphragm and 4 diffuse disease cancer stage (scored from 1 to 4, progressively indicating a worse clinical status of the patient), intent of treatment (adjuvant, new-adjuvant, palliative), toxicity of treatments (scored from 0 (mild) to 5 (death), according to Common Toxicities Criteria for Adverse Events (CTCAE) [20, 21], response to treatment by computerized axial tomography (CAT) (absence of cancer, ongoing, in progress, partial, stable) and adherence to treatment (yes, no).

### 2.4 Statistical Analyses

All data were coded and analyzed using the Statistical Package for Social Sciences (SPSS) version 20. Additional analyses were carried out in R [22], using dedicated packages. All tests were two-tailed, with alpha set at p<0.05. The means with standard deviations were reported for continuous variables. Counts and percentages were reported for categorical variables.

Finite mixture models were applied for testing whether the distribution of TPQ scores in the sample corresponded to a single or a mixture of Gaussian distributions. The analysis was carried out with the mix-tools package running in R [23].

Scales reliability was measured by Cronbach’s alpha. For group comparisons, a rule of thumb assumes that reliability values of 0.70 are considered acceptable and, for subscales, values around 0.60 are considered reasonable [24].

Test-retest reliability of the TPQ was evaluated in a subgroup of 116 participants (out of 263), who were invited to complete the TPQ again after around 3 months. Test-retest stability was assessed with the intraclass correlation coefficient (ICC), with 95% Confidence Interval (CI). The ICC is dimensionless statistics describing the reproducibility of repeated measurements in the same population: ICC values ≥ 0.60 are considered acceptable for clinical use [25].

The Bland and Altman [26] method was also used to assess agreement at re-test for the TPQ. The Bland-Altman plot displays the agreement between the test scores measured at two different assessment points by plotting the difference between test- and retest-scores against the mean of the test- and retest-scores for each participant. Confidence intervals for the mean difference are calculated to determine if the mean difference deviates significantly from zero, which should not. Graphically, the upper and lower limits of agreement are drawn, indicating the range within which 95% of the test scores of two assessments can be expected to vary. The Bland-Altman plot was drawn by adaptation of a pre-existing ad hoc code running in R [27].

## 3 RESULTS

### 3.1 Characteristics of the Sample

The sample included 263 patients with solid or hematological cancers (women = 132; 50.19%). Age in the sample ranged from 19 to 86 years old (mean±sd = 61±14). About half of the sample had a high-school diploma or a university degree (N=139; 52.6%). Most of the participants declared to be married (N=174; 66.1%), with an additional 11 (4.2%) who reported to be divorced and 21 widows/widowers (8%).

The site of the cancer was the gastrointestinal apparatus in 93 patients (35.2%), the blood in 62 patients (23.6%), the gynecological apparatus in 32 patients (12.1%), the breasts in 33 patients (12.5%), the pulmonary apparatus in 16 patients (6.1%), the urogenital apparatus in 17 patients (6.5%), and other sites in the remaining patients (n = 10; 3.9%).

### 3.2 Distribution of the Scores of the TPQ in the Sample

Table **1** summarizes the key descriptive statistics for the TPQ items. Items violated the assumption of normality on the Kolmogorov-Smirnov test (p<0.0001 in all estimations), but deviation from normality was not excessive except for item 2 and item 8, for which kurtosis exceeded the threshold of 2 suggested as a rule of thumb to identify violation of normality [28].

**Fig. (1):**
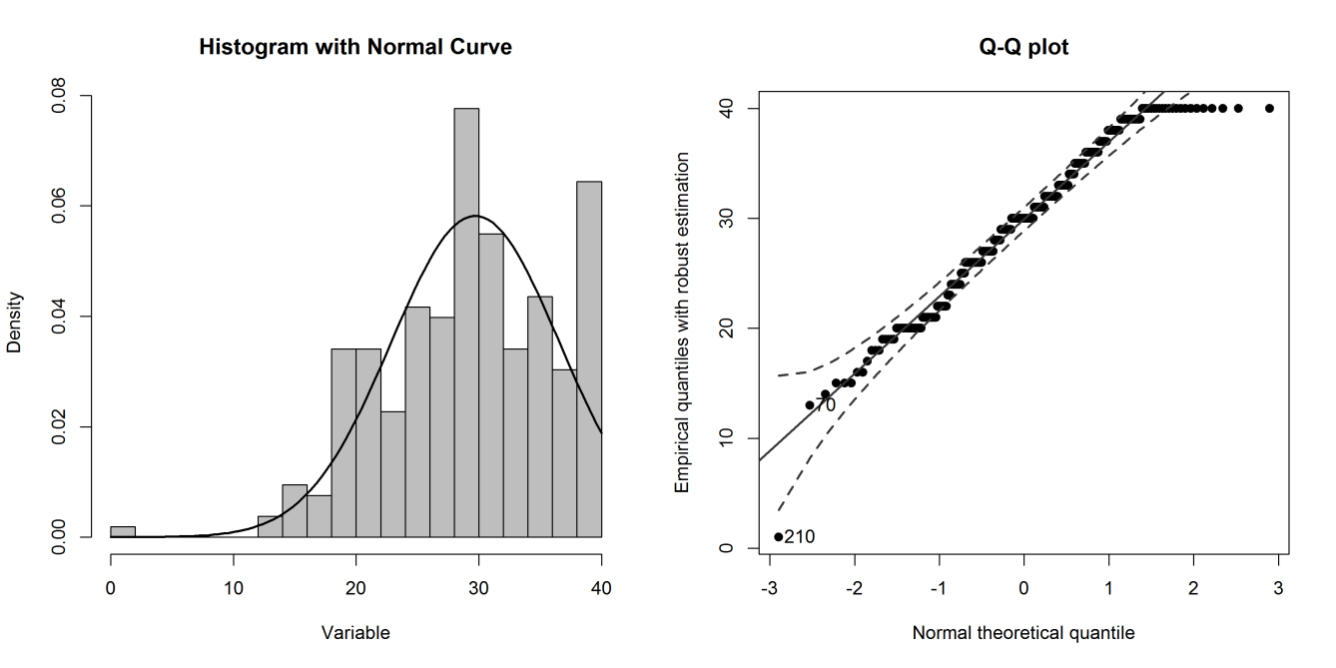
The distribution of TPQ scores in the sample.

Overall, multivariate normality was violated in the sample (Mardia’s test: skew=1463.8, *p*<0.0001; kurtosis=38.0, *p*<0.0001). The distribution of TPQ scores in the sample departed from normality at the very low and high values (Fig. **1**).

Mean in the whole sample was 29.7 (SD = 6.8); median = 30.0 (interquartile range = 9); skewness = -0.46 (standard error of skewness = 0.15); kurtosis = 0.19 (standard error of kurtosis = 0.30).

There were no links of TPQ total scores with sex (Student t*-*test: t=1.33; df=262; p=0.186), age (Pearson’s r=-0.10, p=0.100), or education (F[2;261]=0.70, *p*=0.496).

When compared, patients with solid tumors did not differ from a patient with hematological tumors on TPQ total score (Student t-test: t=-1.16; df 262; p=0.244).

### 3.3 Reliability of the TPQ

Reliability of the total score of the TPQ in the sample was good: Cronbach’s alpha= 0.83 (95%CI: 0.79 to 0.86). Cronbach’s alpha was 0.71 (0.65 to 0.76) for the “program perception” dimension. Reliability of the “staff perception” dimension was fair: Cronbach’s alpha = 0.66 (0.59 to 0.72).

### 3.4 Test-Retest Stability

**Fig. (2):**
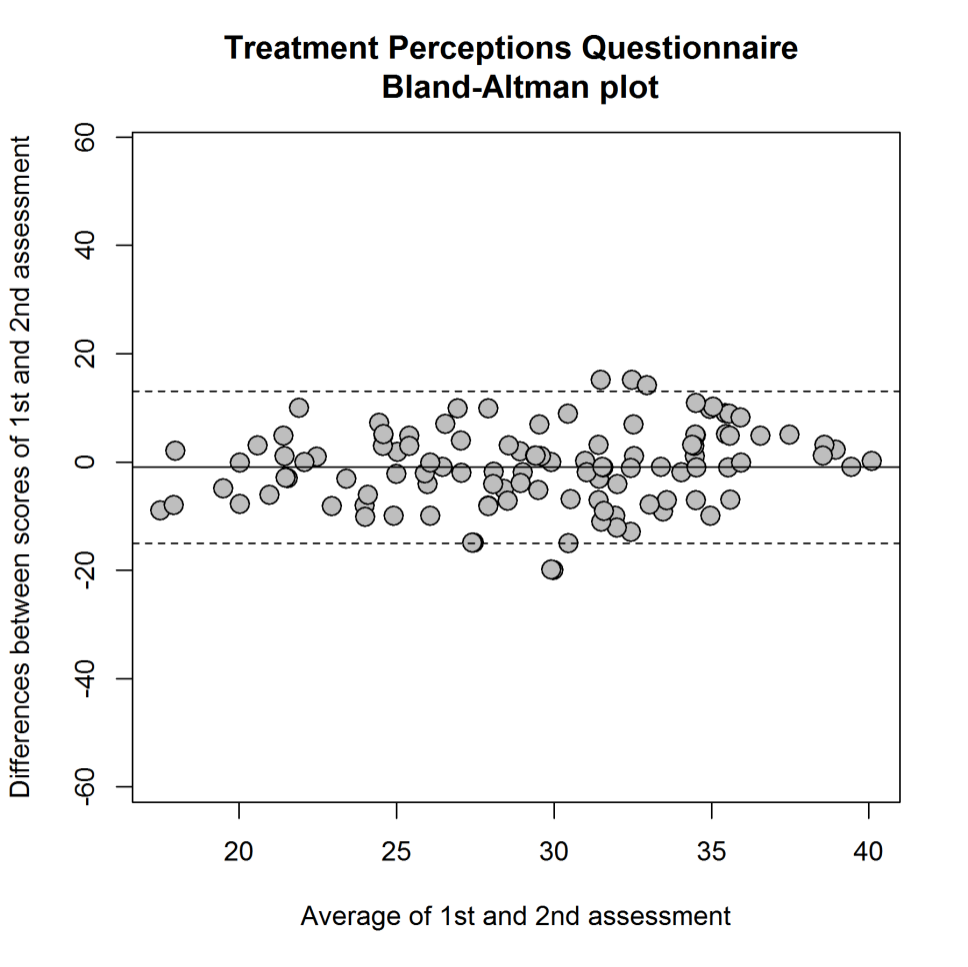
Test-retest reliability of the TPQ – Bland-Altman Plot.

The test-retest reliability of the TPQ, as measured by ICC, was 0.82 (95%CI = 0.77 to 0.87). The mean difference between the first and the second assessment of the TPQ in the 116 participants was 1.0 (SD = 7.1). The 95% CI for the mean difference was – 0.35 to 2.33 (*i.e*., 0 is within the confidence interval. Therefore the mean difference did not differ statistically from 0. By plotting the differences and the means of the two assessments in the Bland-Altman plot, 5 cases only out of 116 (4.3%) were outside the upper and lower limits of agreement (Fig. **2**).

Test- retest stability for program perception (ICC =0.75; 0.68-0.91) was better than for the staff perception (ICC =0.66; 0.57-0.75).

## 4 DISCUSSION

The present study showed good reliability and test-retest stability of the TPQ when it is used in the Oncology field. These findings are comparable to those found in two studies that were used to develop the instrument [18, 19].

The questionnaire contains two five-item sub-scales regarding two dimensions: “staff perceptions” and “care program perception”. The first one concerns beliefs about staff's understanding of the patient's problems, agreement about care aims, availability for talking to, ability to motivate, and professional competence. The second dimension refers to the patient's perceptions about several aspects of the care program, for example, therapeutic content, expectations about treatment efficacy, communication about treatment decision-making, treatment timing, and care program rules. As shown by Cronbach’s alpha index, the reliability of the total score of the TPQ in the sample was satisfactory, as well as for the “program perception” and “staff perception” dimensions. More importantly, test-retest reliability was very good (ICC=0.82) and in the Bland-Altman plot, case were only outside the limits of agreement. Thus, in this sample, the TPQ showed high reliability and stability at retest. As for the subdimensions, we have found that stability for program perception (ICC =0.75; 0.68-0.91) was better than for the staff perception (ICC =0.66; 0.57-0.75). However, in preliminary analyses, we have found that sex, age, or education did not predict the score on TPQ total or subdimensions. Thus, we are not able to define the factors that might influence lower stability of the staff perception.

Unlike the original validation study, the present study shows that “staff perception” dimension had reliability lower than in patients with addiction. This could be because, in the Oncology field, patients’ needs are elevated when it comes to health staff, due to the severity of the illness they suffer from.

Grassi & Nanni [29] underlined the psychosocial dimensions in cancer and implications for care: “the psychology of the cancer patient is the psychology of a person under a special and severe form of stress, during which many fundamental underlying convictions, based on the life-history of the person (and his or her experiences; *e.g*. pattern of the relationship with attachment figures), are brought to the surface”. They [29] suggested the application of the Diagnostic Criteria for Psychosomatic Research (DCPR) in the oncology field to promote an integrated psychiatric/psychosocial approach in oncology [30]. This approach might overcome some reduction of both the Diagnostic Statistical Manual for Mental Disorders (DSM) and the International Classification of Disorders (ICD), which fail to fully describe several psychosocial implications of cancer, such as maladaptive or maladjustment coping styles, psychological symptoms or personality traits that do not meet the full criteria for a specific mental disorder, maladaptive health behaviors, physiological responses to environmental or social stressors.

Indeed, the DCPR considers psychosocial dimensions such as “denial” of having a physical illness that needs treatment, lack of compliance, delayed seeking of medical attention for serious and persistent symptoms, counterphobic behavior as a reaction to the symptoms, signs, diagnosis, or medical treatment of a physical illness [31, 32]. It also considers the “alexithymia”, that is, the inability to use words to describe emotions, the lack of a rich fantasy life with thoughts content associated more with external events, the tendency to describe details instead of feelings, the unawareness of the common somatic reactions that accompany the experience of feelings, and inappropriate outbursts of affective behavior [33-35]. The DPCR also takes into account “demoralization”, which refers to the patient experiences feelings of helplessness, hopelessness, or giving up, due to the patient’s consciousness of failing to meet his or her own expectations or those of others, or being unable to cope with some pressing problem [36, 37]. These factors should be considered because they could influence outcomes of cancer, putting the individual at a higher risk for an adverse outcome, and could impact satisfaction with care.

From this perspective, patient satisfaction can be considered a dynamic process depending on the patient's values, beliefs, expectations, previous health care experiences, and sociodemographic factors. Satisfaction with care is strongly based on the “experiences”, “needs”, “expectations”, what is “important”, “desirable” or “what should be” concerning three main dimensions: the quality of care, health service care, and health staff relational skills. Among the health staff's relational skills are especially relevant empathy, reassurance, emotional and social support given to the patient, and the informal talk with the patient showing the doctor's interest in his or her private matters [6, 11].

The TPQ, as a measure of satisfaction with care, can be considered a person-centered measure able to promote several essential aspects of quality of care, also in the oncology field. One of these aspects regards the intrinsic importance of individuals’ right to be treated with dignity and respect when they are using health services; another one is about the potential association between patients’ satisfaction, improved health care utilization, and other health outcomes [15].

The present study has some limitations. One regards the small sample size, that might have an impact on the power of the statistical analyses; another could be referred to the limited homogeneity of the sample, that includes different kind of patients (in-patients and out-patients) recruited from other types of oncology services, with different cancer diagnoses, so it could be possible that satisfaction with care is a function of these variables. Another limitation regards the use of the Italian translation of the TPQ that even if validated in the field of addiction [19], could impact the reliability and the structure validity of the questionnaire when it is used in a different field, such as oncology, as in the case of the present study. Furthermore, we did not evaluate the convergent and divergent validity of the TPQ with other instruments widely used in the oncology field, such as the EORTC-INPATSAT32 [16], the FAMCARE [17] or the Patient Satisfaction with Cancer Care (PSCC) [9].

Further research is needed to improve the validation of TPQ in the field of oncology, mainly to establish different profiles to the TPQ in people with solid vs blood cancers with regards to other clinical-oncological variables, such as the stage of the illness and the response to treatment.

## CONCLUSION

This pilot study pointed out good reliability and test-retest stability of the TPQ in the oncology field, which was as well as the one observed in the field of addiction. Different from the original validation study, the present one shows that the “staff perception” dimension had reliability lower than in patients with addiction, maybe because, in the oncology field, patients’ needs regarding health staff is elevated, due to the threatening of the illness they suffer from. Overall, the TPQ can be used to assess variations over time of satisfaction with care in patients with oncological diseases.

## Figures and Tables

**Table 1:** Descriptive statistics for the Treatment Perceptions Questionnaire items for the sample (N = 263).

	**Items **	**Dimensions**	**Mean±sd**	**Skewness**	**Kurtosis**
1	The staff have not always understood the kind of help I want*	Staff perception	2.77±1.25	-0.88	-0.35
2	I have been well informed about decisions made about my treatment	Program perception	3.31±0.82	-1.82	4.74
3	The staff and I have had different ideas about what my treatment objectives should be*	Staff perception	2.62±1.34	-0.75	-0.72
4	There has always been a member of staff available when I have wanted to talk	Staff perception	3.20±0.97	-1.46	1.99
5	The staff have helped to motivate me to sort out my problems	Staff perception	3.15±0.94	-1.30	1.75
6	I have not liked all of the treatment sessions I have attended*	Program perception	2.69±1.25	-0.79	-0.44
7	I have not had enough time to sort out my problems*	Program perception	2.68±1.27	-0.85	-0.38
8	I think the staff have been good at their jobs.	Staff perception	3.40±0.81	-1.84	4.58
9	I have received the help that I was looking for	Program perception	3.26±0.89	-1.57	2.96
10	I have not liked some of the treatment rules or regulations*	Program perception	2.70±1.16	-0.74	-0.43

*Legend* *Reverse scoring: sd= standard deviation

## Data Availability

Not applicable.

## References

[1] Bleich S.N., Ozaltin E., Murray C.K. (2009). How does satisfaction with the health-care system relate to patient experience?. Bull. World Health Organ..

[2] Bjertnaes O.A., Sjetne I.S., Iversen H.H. (2012). Overall patient satisfaction with hospitals: Effects of patient-reported experiences and fulfilment of expectations.. BMJ Qual. Saf..

[3] Bogner H.R., de Vries McClintock H.F., Hennessy S., Kurichi J.E., Streim J.E., Xie D., Pezzin L.E., Kwong P.L., Stineman M.G. (2015). Patient satisfaction and perceived quality of care among older adults according to activity limitation stages.. Arch. Phys. Med. Rehabil..

[4] Bauer U.E., Briss P.A., Goodman R.A., Bowman B.A. (2014). Prevention of chronic disease in the 21st century: Elimination of the leading preventable causes of premature death and disability in the USA.. Lancet.

[5] Anderson G., Knickman J.R. (2001). Changing the chronic care system to meet people’s needs.. Health Aff. (Millwood).

[6] Hayran O., Özer O. (2018). Organizational health literacy as a determinant of patient satisfaction.. Public Health.

[7] Moreno P.I., Ramirez A.G., San Miguel-Majors S.L., Fox R.S., Castillo L., Gallion K.J., Munoz E., Estabrook R., Perez A., Lad T., Hollowell C., Penedo F.J. (2018). Satisfaction with cancer care, self-efficacy, and health-related quality of life in Latino cancer survivors.. Cancer.

[8] Lin J., Hsieh R.K., Chen J.S., Lee K.D., Rau K.M., Shao Y.Y., Sung Y.C., Yeh S.P., Chang C.S., Liu T.C., Wu M.F., Lee M.Y., Yu M.S., Yen C.J., Lai P.Y., Hwang W.L., Chiou T.J. (2020). Satisfaction with pain management and impact of pain on quality of life in cancer patients.. Asia Pac. J. Clin. Oncol..

[9] Jean-Pierre P., Fiscella K., Winters P.C., Post D., Wells K.J., McKoy J.M., Battaglia T., Simon M.A., Kilbourn K., Patient Navigation Research Program Group (2012). Psychometric development and reliability analysis of a patient satisfaction with interpersonal relationship with navigator measure: A multi-site patient navigation research program study.. Psychooncology.

[10] Rechel B., Grundy E., Robine J.M., Cylus J., Mackenbach J.P., Knai C., McKee M. (2013). Ageing in the European Union.. Lancet.

[11] Zawisza K., Galas A., Tobiasz-Adamczyk B. (2020). Factors associated with patient satisfaction with health care among Polish older people: results from the polish part of the COURAGE in Europe.. Public Health.

[12] Piazza M.F., Galletta M., Portoghese I., Pilia I., Ionta M.T., Contu P., Mereu A., Campagna M. (2017). Meeting psychosocial and health information needs to ensure quality of cancer care in outpatients.. Eur. J. Oncol. Nurs..

[13] Hekkert K.D., Cihangir S., Kleefstra S.M., van den Berg B., Kool R.B. (2009). Patient satisfaction revisited: A multilevel approach.. Soc. Sci. Med..

[14] Perkins HS, Freed AA, Cortez JD, Hazuda HP (2020). Inpatient culture and satisfaction with care: A novel perspective.. Am J Med Sci..

[15] Larson E., Sharma J., Bohren M.A., Tunçalp Ö. (2019). When the patient is the expert: Measuring patient experience and satisfaction with care.. Bull. World Health Organ..

[16] Brédart A., Bottomley A., Blazeby J.M., Conroy T., Coens C., D’Haese S., Chie W.C., Hammerlid E., Arraras J.I., Efficace F., Rodary C., Schraub S., Costantini M., Costantini A., Joly F., Sezer O., Razavi D., Mehlitz M., Bielska-Lasota M., Aaronson N.K., European Organisation for Research and Treatment of Cancer Quality of Life Group and Quality of Life Unit (2005). An international prospective study of the EORTC cancer in-patient satisfaction with care measure (EORTC IN-PATSAT32).. Eur. J. Cancer.

[17] Lo C., Burman D., Rodin G., Zimmermann C. (2009). Measuring patient satisfaction in oncology palliative care: psychometric properties of the FAMCARE-patient scale.. Qual. Life Res..

[18] Marsden J, Duncan S, Gossop M, Rolfe A, Bacchus L, Griffiths P, Clarke K, Strang J (2000). Assessing client satisfaction with treatment for substance use problems and the development of the treatment perceptions questionnaire (TPQ).. Addict Res..

[19] Marsden J, Nizzoli U, Corbelli C, Margaron H, Torres M, Prada De Castro I, Stewart D, Gossop M. (2000). New European instruments for treatment outcome research: Reliability of the maudsley addiction profile and treatment perceptions questionnaire in Italy, Spain and Portugal.. Eur Addict Res..

[20] Addiction Research Unit Maudsley Hospital/Institute of Psychiatry, UK.

[21] Basch E., Reeve B.B., Mitchell S.A., Clauser S.B., Minasian L.M., Dueck A.C., Mendoza T.R., Hay J., Atkinson T.M., Abernethy A.P., Bruner D.W., Cleeland C.S., Sloan J.A., Chilukuri R., Baumgartner P., Denicoff A., St Germain D., O’Mara A.M., Chen A., Kelaghan J., Bennett A.V., Sit L., Rogak L., Barz A., Paul D.B., Schrag D. (2014). Development of the National Cancer Institute’s patient-reported outcomes version of the common terminology criteria for adverse events (PRO-CTCAE).. J. Natl. Cancer Inst..

[22] R Core Team A language and environment for statistical computing.. R Foundation for Statistical Computing, Vienna, Austria.

[23] Benaglia T., Chauveau D., Hunter D.R., Young D. (2009). Mixtools: An R package for analyzing finite mixture models.. J. Stat. Softw..

[24] Nunnally J.C., Bernstein I.H. (1994). Psychometric theory..

[25] Brennan P., Silman A. (1992). Statistical methods for assessing observer variability in clinical measures.. BMJ.

[26] Bland J.M., Altman D.G. (1999). Measuring agreement in method comparison studies.. Stat. Methods Med. Res..

[27] Mateos J.M. (2018). Bland-Altman plot, R code.. GitHub Gist.

[28] Trochim W.M., Donnelly J.P. (2006). The research methods knowledge base..

[29] Grassi L., Nanni M.G. (2013). Beyond psychiatric classification in oncology: Psychosocial dimensions in cancer and implications for care.. Psycho-oncol..

[30] Porcelli P., Rafanelli C. (2010). Criteria for psychosomatic research (DCPR) in the medical setting.. Curr. Psychiatry Rep..

[31] Fava G.A., Fabbri S., Sirri L., Wise T.N. (2007). Psychological factors affecting medical condition: A new proposal for DSM-V.. Psychosomatics.

[32] Sirri L., Fava G.A. (2013). Diagnostic criteria for psychosomatic research and somatic symptom disorders.. Int. Rev. Psychiatry.

[33] Sancassiani F., Preti A., Cacace E., Ruggiero V., Testa G., Romano F., Carta M.G. (2019). Alexithymia and sense of coherence: Does their impact on fibromyalgia suggest new targets for therapy?. Gen. Hosp. Psychiatry.

[34] Porcelli P., Guidi J., Sirri L., Grandi S., Grassi L., Ottolini F., Pasquini P., Picardi A., Rafanelli C., Rigatelli M., Sonino N., Fava G.A. (2013). Alexithymia in the medically ill. Analysis of 1190 patients in gastroenterology, cardiology, oncology and dermatology.. Gen. Hosp. Psychiatry.

[35] Carta M.G., Sancassiani F., Pippia V., Bhat K.M., Sardu C., Meloni L. (2013). Alexithymia is associated with delayed treatment seeking in acute myocardial infarction.. Psychother. Psychosom..

[36] Belvederi Murri M., Zerbinati L., Ounalli H., Kissane D., Casoni B., Leoni M., Rossi G., Dall’Olio R., Caruso R., Nanni M.G., Grassi L. (2020). Assessing demoralization in medically ill patients: Factor structure of the Italian version of the demoralization scale and development of short versions with the item response theory framework.. J. Psychosom. Res..

[37] Grassi L., Spiegel D., Riba M. (2017). Advancing psychosocial care in cancer patients.. F1000 Res..

